# Day centres for older people - attender characteristics, access routes and outcomes of regular attendance: findings of exploratory mixed methods case study research

**DOI:** 10.1186/s12877-020-01529-4

**Published:** 2020-05-04

**Authors:** Katharine Orellana, Jill Manthorpe, Anthea Tinker

**Affiliations:** 1 NIHR Policy Research Unit on Health and Social Care Research, The Policy Institute, King’s College London, Strand Campus, London, WC2R 2LS UK; 2 Institute of Gerontology, King’s College London, Strand Campus, Strand Campus, London, WC2R 2LS UK

**Keywords:** Day centres, Older people, Ageing, Frailty index, Social isolation, Social support, Group activity, Social prescription, Outcomes

## Abstract

**Background:**

Social prescribing is encouraged to promote well-being, reduce isolation and loneliness. Traditional, generalist day centres for older people could be suggested by social prescribing, but little is known about their clientele or their outcomes. As part of a larger study of the role, outcomes and commissioning of generalist English day centres for older people, the characteristics of attenders at 4 day centres, their reasons for attendance and outcomes were explored.

**Methods:**

This mixed-methods study used qualitative interviews and standardised tools within an embedded multiple-case study design. Semi-structured interviews with older day centre attenders (*n* = 23, 62% of eligible attenders) of 4 day centres in south-east England, recruited purposively to reflect organisational differences, were analysed.

**Results:**

Participants reported non-elective withdrawal from socialisation following health or mobility decline, or losses. Apart from living arrangements and marital status, attenders’ profiles differed between centres. Access had been mostly facilitated by others. Day centre attendance enhanced quality of life for this group of socially isolated people with mobility restrictions and at risk of declining independence and wellbeing. The positive impact on attenders’ social participation and involvement and on meaningful occupation was significant (*p*-value < 0.001, 99% CI), with an average ASCOT gain score of 0.18. Ten outcome themes were identified.

**Conclusion:**

Outcomes of day centre attendance are those targeted by social care and health policy. Centres were communities that ‘enabled’ and offset loss or isolation, thus supporting ageing in place through wellbeing and contributed something unique to their attenders’ lives. By monitoring attenders’ health and wellbeing and providing practical support, information and facilitating access to other services, centres offered added value. Attendance needs to be set in the context of other social engagement and care provision which may not overlap or duplicate centre support. Professionals may wish to explore the benefits of social prescriptions to day centres but should map local centres’ provision, engage with their organisers, and seek information on attenders, who may differ from those in this study.

## Background

Day centres for older people are community building-based services that provide care and/or health-related services and/or activities specifically for older people who are disabled and/or in need. Attendance can be for a whole or part of a day and cover any number of days. Centres offer a wide variety of programmes that may be considered ‘preventive’ of decline or ill-being. Public funding reductions [1] have led to fewer older people with high needs being eligible for state funded social care in England. Many day centres, particularly those offering ‘low-level’ support (support to people without high personal care or physical support needs), have closed or been decommissioned [2], despite evidence that some older people would like to attend them [3]. However, about 59,000 older people using local authority provided or commissioned community services attend day centres [4]. Within this context of change and ongoing efforts to integrate health and social care, the future of day centres is uncertain.

Although evidence-based commissioning is encouraged within English social care [5] and the NHS [6], some interventions, particularly, ‘preventive’ services, have an under-developed evidence base [7]. Evidence generation for day centres is further complicated by their heterogeneity; centres may be owned by different types of provider, operate in different types of building, and may differ in size, target clientele and funding sources. Moreover, national data are difficult to obtain in England as day centres are not regulated. Outcomes data are increasingly relevant, with Outcomes Frameworks for health and social care introduced in 2014–15 [8]. Reflecting the policy direction of the NHS Five Year Forward View [9] and Care Act 2014 [10], these Frameworks share the goals of enhancing the quality of life of people with care and support needs, delaying and reducing the need for care, ensuring positive care experiences and safeguarding from abuse.

Much early research about day centres was undertaken in different policy and funding contexts or concerned centres specialising in dementia care [11]; the last detailed study of day care in England and Wales being published in 1989 [12]. However, by applying National Institute for Health and Care Excellence (NICE) criteria, English day centres having been judged cost-effective [13]. A review of the 2005–17 UK and non-UK literature [11], undertaken for the present study of four English day centres for older people, identified a lack of research about day centres as whole services and evidence gaps concerning what they offer, who uses them and why, what outcomes are experienced and how centres are perceived by their various stakeholders and potential users [11]. Ellen et al.’s review of the literature about day centre outcomes called for better understanding of people’s characteristics when they start attending a day centre and factors relating to their access [14]. This might inform funding and referring decisions by, for example, primary care professionals.

Wye et al. [15] reported that clinical commissioners seek evidence and information mainly from trusted health-related sources (for example, the National Institute for Health and Care Excellence (NICE), the King’s Fund and NHS Improving Quality), their own experience or local contacts. Few sources of academic research are consulted as these are considered to lack rich contextual data. Real life examples which provide context for impact make an important contribution to their knowledge and decision-making. Mixed methods are increasingly being used in health services research to provide such evidence to ‘*capture the experiences, emotions, and motivations of people providing and receiving health care, as well as the objective conditions of care delivery*’ (i.e. ref [16], page 2126).

This paper seeks to address the limitations that Wye et al. [15] identified as present in information available to clinical commissioners by illustrating what can be learned from profiling the attenders of 4 day centres and the outcomes they experienced, and contextualising these within current policy and demographic data. It reports frequency and reasons for attendance and access in the context of life with needs for care and support. These data contextualise the outcomes experienced, also reported here, which, like other studies [13], indicate the generally positive outcomes for attenders. We argue that such information could be analysed in other areas to improve strategic decisions about service commissioning and referral pathways for older patients with long-term conditions and limited social support.

## Methods

This paper draws on findings of a three-year study [17] that aimed to paint an in-depth, rich and contemporary picture of 4 day centres for older people by investigating what they offered, who used them, why and how, what they contributed to the lives of those involved in them. At macro level, it further explored professional perceptions, and centres’ relationships with local health and care services, and the potential utility of collecting data about attenders using standardised measures. The study was of ‘generalist’ day centres, those not offering specialist care to a specific group, such as people living with dementia or stroke survivors, because more older people live without dementia than with it [18]. In the UK, there has been substantial investment in dementia research, including studies of day services for older people living with dementia [19]. The study conceived day centres as a potential service to support people to remain in their home with changing needs [20, 21]. On the one hand, ageing in place is beneficial for quality of life, more cost-effective for public funds [22] and addresses the increased support needs associated with growing numbers of oldest-old people [23]. On the other, very old people (≥85 years) spend an average of 80% of their time at home [24]; staying at home for most of the time due to health constraints may lead to poor wellbeing [25], and social isolation has a negative health impact [26].

An embedded multiple-case study approach [27] was taken, with day centres as cases and individual participant groups as embedded cases. According to Yin [27], case studies may be descriptive, exploratory, explanatory, comparative or evaluative. This study is both descriptive, as it aims to present characteristics, and explanatory, as it is concerned with explanations or analysis. Taking a pragmatic approach [28, 29] prompted mixed data collection methods. Neither method was privileged as combining data collection methods is argued to be more effective when each is valued equally as findings triangulate with each other, cover the other’s ‘blind spots’ and ‘*provide a ‘fuller’ picture of the phenomenon being studied.’* (i.e. ref [30], page 40). Drawing on the strengths of different approaches [28] enabled gaining in-depth insights into day centres’ role and ascertaining measurable impact and maximises the breadth and depth of evidence [31, 32]. Qualitative research does not seek a representative sample and does not aim for generalisability; instead it seeks diverse and rich responses.

The first author, a female social gerontologist with 15 years of experience in the charitable ageing sector and long-standing interest in day centres, undertook weekly visits over 13 months to 4 day centres (14 weeks at each centre) during which she spoke to attenders, and interviewed 69 participants (older attenders (*n* = 23), attenders’ family carers (*n* = 10), day centre managers, frontline staff and volunteers (*n* = 23) and local authority staff responsible for service commissioning or referral (*n* = 13)). A donation of £100 was given to each centre following fieldwork, a strategy intended to enable all attenders, carers and personnel at the centres to feel appreciated.

### Samples

#### Day centres

Day centres were recruited against a typological matrix of characteristics (provider, building designation, admission criteria, attender numbers and target users). This maximum variation approach [33, 34] aimed to increase the heterogeneity of centres and individuals involved with them. Centres were in rural and urban areas with differing population characteristics but, due to time and funding constraints, were in South-East England and London.

Centres were operated by one local authority (DCLA), one housing association (DHCA), and two voluntary sector providers (DCV1, DCV2). The former two operated for 5 days and accepted local authority referrals only. The latter two operated for one and 2 days. One was openly accessible for people aged ≥60 years and housebound, those who were socially isolated and who may be in receipt of care from statutory or voluntary agencies, but not for people needing lifting, personal care or nursing, or people needing specialist care for mental illness. The other accepted both open and local authority referrals of people who were socially isolated with transport needs but not requiring personal care assistance.

Attendance on research days over the research period varied: DCHA 9–12 older people per day; DCLA 22–28 older people per day; DCV1 12–14 older people per day; DCV2 6–11 older people per day.

#### Attenders

Daily capacity restrictions led to setting maximum recruitment quotas of 10 per centre. At the start of visit periods, attenders falling outside inclusion criteria of attending on the researcher’s ‘visiting’ day, with the ability to understand hypothetical situations and give informed consent were identified with managers and/or frontline staff. Weekly visits to centres enabled the researcher to build a rapport with attenders which facilitated further decisions about eligibility during informal conversations. Where there was lack of clarity regarding a potential participant’s (in)eligibility, this was discussed further with staff. Efforts were made to be as inclusive as possible by offering the opportunity for paired interviews, with a support person, where communication assistance was needed (for example, due to disability or language) or potential participants wished for support, and by using showcards in interviews. All those eligible to participate were given a study Information Sheet and invited to participate.

Just over half of all attenders observed to attend the four centres on research days during the visit period (*n* = 68) met inclusion criteria (*n* = 37, 54%). Of those eligible, almost two-thirds (*n* = 23, 62%) participated, equating to one third of all attenders (34%). Eligible non-participants (*n* = 14, 38% of 37 eligible) refused due to health or other problems occupying their minds and depleting their energy (*n* = 8), were persistently unavailable (*n* = 4), ill (*n* = 1) or died (*n* = 1). Not meeting the inclusion criteria (*n* = 31, 46%) was for reasons of severe dementia-related cognitive impairment or memory loss (*n* = 28), not being able to think hypothetically due to learning disability (*n* = 2) and severe stroke-related speech impairment (*n* = 1).

### Data collection

The first author conducted individual face-to-face semi-structured interviews, with prompts, between September 2015 and December 2016. Qualitative interviews followed a thematic guide which comprised questions concerning what a ‘usual week’ looked like; why, and how, they started attending the day centre and any benefits they had experienced, including whether attending the day centre added anything to their life they felt they would not otherwise have experienced (i.e. something unique) (see Additional File 1 for interview questions). Outcomes data were also gathered by using the Adult Social Care Outcomes Toolkit (ASCOT INT4) [33] validated instrument. Socio-demographic data were then collected, with participants’ health, wellbeing and social networks characteristics gathered using validated tools: the Edmonton Frail Scale (EFS), the Short Warwick-Edinburgh Mental Wellbeing Scale (SWEMWBS) and the Practitioner Assessment of Network Type (PANT). Tool use was registered and the PANT training pack [34] obtained.

The qualitative parts of interviews took place in participants’ homes (*n* = 16) or in private spaces at day centres (e.g. meeting rooms, vacant hairdressing salon) (*n* = 7) and lasted, on average, 42 min. One attender’s support worker was present at her interview. Qualitative interviews were recorded and transcribed externally. Participants were not requested to comment on transcripts.

#### Adult Social Care Outcomes Toolkit (ASCOT) INT4 (v3.3)

ASCOT INT4 is a subjective multi-domain, cost-utility tool used to measure social care-related quality of life (SCRQoL) outcomes [35]. By measuring relative value of services to the individual [13], it enables fairer comparison of effectiveness between services. Administered once, it measures current SCRQoL and expected SCRQoL in the absence of service where nothing takes its place. SCRQoL gain scores, indicating service impact on quality of life, are calculated by deducting expected from current scores. In eight domains (see Table 1), answers categorise needs into high needs, some needs, no needs, and ideal state. Scores are preference-weighted, based on responses to a Best-Worst scaling approach and a time trade-off exercise tested with a non-service using population and service users, with greater weight given to domains reported as most important [36]. The higher the gain score, the bigger a positive difference day centre attendance makes to SCRQoL. Weighted scores may range from − 0.17 to 1, where < 0 is worse than dead, 0 is the equivalent of being dead and 1 is an ideal situation. Unweighted scores may range from 0 to 100. It has good construct validity with older people [37] and its subjective nature and preference weightings mean greater validity in measuring the effects of social care services than EQ5D [38]. Cost-effectiveness can be ascertained if service costs are known. Administration was relatively straightforward, lasting, on average, 15 min.

**Table 1:** ASCOT INT4 Domains (summarised from Netten et al. 2011:3, ref. [36])

Domain		The person…
1)	*Accommodation cleanliness and comfort*	…feels their home environment, including all rooms, is clean and comfortable
2)	*Personal cleanliness and comfort*	…feels personally clean and comfortable and looks presentable or, at best, is dressed and groomed in a way that reflects personal preferences
3)	*Food and drink*	…feels they have a nutritious, varied and culturally appropriate diet with enough food and drink that they enjoy at regular and timely intervals
4)	*Personal safety*	…feels safe and secure, meaning being free from fear of abuse, falling or other physical harm both inside and outside the house
5)	*Social participation and involvement*	…feels content with their social situation (i.e. sustenance of meaningful relationships with friends, family and feeling involved or part of a community should this be important)
6)	*Occupation*	…sufficiently occupied in a range of meaningful activities
7)	*Control*	…can choose what to do and when to do it, having control over daily life and activities
8)	*Dignity*	Negative and positive psychological impact of support and care on personal sense of significance.

#### Edmonton Frail Scale (EFS)

This includes nine questions on general health, functional independence, social support, medication use, nutrition, mood, continence, that are supplemented by the clock-drawing test, for cognition, and the Timed Up and Go (TUG) test, for functional performance. This objective screening tool reflects frailty’s multi-dimensional, unstable and heterogeneous nature [39], including social support. It has been tested with older people and validated as a reliable and feasible tool for use by non-geriatricians [39]. As frailty is associated with adverse health outcomes, ascertaining frailty levels can assist care planning [40]. Scores (0–17) are converted to categories, with higher scores indicating greater frailty: No frailty (0–4), Apparently vulnerable (5–6), Mild frailty (7–8), Moderate frailty (9–10) and Severe frailty (≥11).

#### Short Warwick-Edinburgh Mental Wellbeing Scale (SWEMWBS)

This 7-item scale measures feelings and functioning (not illness and disorder) using positively-worded statements [41]. Its use of raw scores (7–35) and metric (transformed) scores (7.00–35.00) enable it to be used as an interval scale for psychometric analysis [42]. Higher scores indicate higher wellbeing.

#### Practitioner Assessment of Network Type (PANT)

An eight-question objective tool measuring social support networks in populations aged 50 or older [43], PANT covers level and frequency of contact and physical distance. Likelihood of need for formal services can be predicted based on network typology as each is associated with specific risks [44]. People with Locally Self-Contained networks are more likely to be isolated as people rely on neighbours, lead private lives and have little community involvement; they are, however, in contact with family over 50 miles away. Family Dependent and Private Restricted networks carry greater risks of depression, loneliness and other mental ill-health. People with the former type rely on local family, but also have some neighbour contact and some community group involvement. People with the latter have no local family, no local informal support and little community contact, but people may rely on distant family. Locally Integrated or Wider Community-focused networks are the strongest types. In the former, informal help is exchanged between family, friends and neighbours, and people have community group involvement. In the latter, people do not have local family, but exchange informal help with friends and are in contact with family over 50 miles away; there is some neighbour involvement and high community group involvement.

### Data analysis and presentation

Raw data for PANT and ASCOT were entered into two specially-designed tools: an SPSS syntax file computed PANT network type [45] and an ASCOT Microsoft Excel data entry tool (v2.9) computed unweighted and weighted scores [46]. EFS and SWEMWBS scores were calculated manually using guidance. After removing identifiers, qualitative data were entered into NVIVO (version 11) [47], a Computer-Assisted Qualitative Data Analysis Software. Individuals’ attributes and validated scale scores were assigned to participants’ NVIVO records to enable interrogation of data by day centre and participant characteristics [48].

Attenders’ usual weeks were recorded on ‘maps of the week’ which were entered into MS Excel, colour-coded by type of activity, then manually analysed for descriptive purposes. Maps were not analysed for patterns due to small numbers.

Analysis of qualitative data was inductive, iterative and thematic [49]. The first author undertook coding and analysis, supported by team meetings with the second and third authors in which data saturation and analysis were discussed and verified. Familiarisation with data involved listening to audio recordings, correcting and anonymising transcripts while concurrently noting down codes. These codes were grouped into themes and sub-themes, under each research question, and entered into NVIVO. Iterative lumper coding [48] was then undertaken. This included reviewing and re-reviewing coded data, adding, amending, merging and deleting codes as necessary (see Additional File 2 for the coding frame). Text was simultaneously coded where relevant to more than one code [50]. Qualitative data was coded after calculating ASCOT scores, but ASCOT domains did not influence decisions about qualitative outcome themes. Cross-case analyses of individual participant group data were undertaken; themes were identified across the different day centres and participant sub-groups [51].

Mixed methods data presentation is potentially problematic, affected, for example, by word limits [52, 53]. Given that qualitative and quantitative findings about outcomes are complementary and triangulate with each other, these are presented together. Pseudonyms are used in this paper.

### Stakeholder involvement

A Study Advisory Group supported the study and met three times. Members, all with experience of day centres, were consulted about study materials [51] and interpretation of findings. A separate Advisory Group that acts as a critical friend to the researcher’s host Unit also provided feedback on study materials and the first author’s interpretation of findings at two of its meetings. Case study site representatives (six local authority staff responsible for service commissioning or referral and 2 day centre assistant/managers) attending a workshop were also consulted about these.

## Day centre attenders’ characteristics, reasons for attending, access to centres and contexts of attendance

### Participant characteristics

Most attender participants were widowed, divorced or single, and two-thirds lived alone (see Table 2). Their average age was 83.3 years (range 68–101 years), three-quarters were women and one-quarter were educated beyond secondary school. All identified as heterosexual and as not having changed gender. Three-quarters held religious beliefs. Ethnic minority groups accounted for a quarter of the total number, but were only in two of the four centres, reflecting local demography. Indicative of relative deprivation, two-thirds lived in rented homes while just under one-third were owner-occupiers. Although similar numbers received means-tested benefits as those who did not, almost two-thirds self-funded their centre attendance (*n* = 14). Self-funders included people whose financial assessment, after being assessed as eligible for services, required them to pay for themselves (*n* = 3) and people not mentioning any assessment (*n* = 11). Others reported being fully local authority (LA) (means-tested) funded (*n* = 6), sharing payment with the LA (*n* = 1) or being unaware of who paid (*n* = 2).

**Table 2:** Attender participants’ characteristics (*n* = 23)

Characteristics		Number
**Age**	65–69	2
	70–74	1
	75–79	4
	80–84	6
	85–89	5
	90–94	3
	≥95	1
	Not given	1
**Sex, gender identity and sexual orientation**	Male	5
	Female	18
	Gender same as birth	23
	Heterosexual	23
**Marital status**	Widowed/ surviving partner	15
	Separated/ divorced	5
	Never married	1
	Married	2
**Living arrangements**	Alone	15
	With adult children/other family	6
	With spouse	2
**Accommodation**	Owner-occupied	7
	Rented - privately	2
	Rented - LA/housing association	14
**Means-tested financial help**	Pension Credit & Housing Benefit	9
	Pension Credit	2
	No means-tested financial help	10
	Does not know	2
**Education**	Secondary & further education	6
	Completed secondary school	15
	Did not complete secondary	2
**Ethnicity**	White British /English	16
	White - any other	1
	Black Caribbean	5
	Asian - any other	1
**Religion**	Christian	17
	Judaism	1
	Atheism/no religion or belief	5
**Health conditions or disabilities**	Have a health condition or disability	23
	General learning disability	2
	Mental health condition	2
	Blind/partially sighted - uncorrected by glasses	3
	Other (e.g. hip replacements, stroke, thrombosis)	5
	Deafness or serious hearing impairment	8
	A physical disability or mobility difficulties	17
	A long-standing illness or health condition	17
**Edmonton Frail Scale (EFS)**	Severe frailty (11–17)	5
	Moderate frailty (9–10)	3
	Mild frailty (7–8)	4
	Apparently vulnerable (5–6)	9
	No frailty (0–4)	2
**Practitioner Assessment of Network Type (PANT)**	Locally integrated	8
	Locally self-contained	8
	Local family-dependent	3
	Private-restricted	3
	Borderline family-dependent & locally self-contained	1
	Wider community-focussed	0
**Short Warwick-Edinburgh Mental Wellbeing Scale (SWEMWBS) (*n*** **= 22)**	Raw score range: 18–35 (mean 27.18) Metric score range: 17.43–35 (mean 24.78)	
	Good wellbeing (> 1 SD above mean)	4
	Average wellbeing (±1 SD of mean)	13
	Poor wellbeing (< 1 SD below mean)	5

All reported having health conditions or disabilities that impacted greatly on their life, with half reporting at least two forms of these. All, except two, had some levels of Apparent Vulnerability or frailty when measuring general health status with the EFS. Levels of health conditions were under-reported by attenders; some did not report certain health conditions in the interview that the researcher was later made aware of in family carer interviews, by centre staff during discussions or by the attenders themselves, for example, that one participant had dementia and another had terminal cancer and had survived a stroke. Three-quarters of attenders had average or good wellbeing as measured by the SWEMWBS.

Two-thirds were at greater risk of isolation, depression, loneliness and other mental ill-health because of their PANT social network type. Only one-third had a stronger social network type.

Apart from marital status and living arrangements, the profiles of attenders varied between centres. DCLA was most age-diverse. DCHA was the least gender diverse. People who could be classified as having Severe Frailty were highest in number at DCHA, despite its younger profile; levels of Apparent Vulnerability were matched across the remaining three centres. DCHA and DCLA attenders had the highest levels of mobility difficulties and more long-standing health conditions, while deafness was most prevalent at DCV1 and DCV2. Owner-occupiers prevailed at DCV1 and DCV2 and renters at DCLA and DCHA.

### Reasons for attending

Circumstances when starting to attend a day centre mainly related to loss and a desire for something different in life. These were classified into six themes: 1) social isolation (mainly due to bereavement or having lost existing social networks), 2) loss of mobility (declining physical health, sometimes suddenly, or no longer driving their car), 3) activity-related (stopping attending another day centre or club due to closure or changed entry criteria, stopping volunteering or retirement, wanting ‘something to do’ for stimulation or a change, or ‘somewhere to go’), 4) mental health or emotional problems (feeling depressed or very low, lonely, having lost confidence or reporting a diagnosed anxiety disorder), 5) feeling ‘stuck’ at home or not getting out enough, and 6) carer-related (recognising the need for family to have a break, feeling isolated as a spousal carer, accompanying cared-for to a day centre).

Principal motivations for starting to attend a day centre reflected these circumstances in that participants had wanted social contact, something to do, to get out of their home or to improve their mental health. Additional motivations were to meet goals for better physical health through exercise and meals, to improve mental health and to accompany a partner for whom the participant provided care. Although length of attendance ranged from a few months to decades, these motivations are likely to reflect attenders’ marital status, living arrangements and health. Behind one principal motivation for attendance were different clusters of circumstances which interacted and overlapped, often triggered by an event or a series of interlinked events. Table 3 illustrates the complexity of two attenders’ circumstances when they started thinking about attending a day centre.

**Table 3:** Examples of two pseudonymised attenders’ circumstances prior to attending a centre

Attender	Circumstances
**Ruth**	Depression and loss of confidence following widowhood led Ruth to stop driving her car. Doing so resulted in lost social networks and activities. The situation worsened after a period of illness which left her unable to walk to a volunteering commitment. She lived alone and wanted social contact which she had lost after stopping driving.
**Wilma**	Wilma fell ill immediately after being widowed. After a stroke, she lost physical mobility and, consequently, social networks, despite her son and his family living in the same house. Wilma wanted a change of environment but was unable to get out of the house without help.

### Accessing day centres

Despite having lived in their areas for an average of 40.5 years (range 10–84 years), only two had known of their current centre. Half (*n* = 11) had never heard of day centres before attending one.

Attenders had accessed their day centres through different routes. Almost half (*n* = 11) reported starting attendance following contact with social care or health professionals (social workers = 7, district nurse = 1, hospital rehabilitation service = 1); the tenth was unsure of the professional’s identity and the eleventh had spoken to a GP, then a social worker. Six had found out about centres from family (*n* = 4), from its manager who was an acquaintance (*n* = 1), and by another attender’s recommendation (*n* = 1). Some paths were less straightforward. Two had been told by a local councillor (politician) and a GP, and had subsequently had social work involvement. The former received a social worker assessment after starting to attend. The latter, after a ‘bad’ first experience at another centre she had been referred to by her GP, had asked her family to find an alternative centre. In the event, the LA-provided home care worker had linked them to a social worker who arranged the current centre place. Two had proactively approached social workers about centres. Four participants were unsure how they found out about their centres.

### Contexts of current day centre attendance

Average weekly centre attendance was for 1.8 days. Overall, just over half (*n* = 13, 56%) attended once weekly, one-third (*n* = 8, 35%) for two (*n* = 4) or three (*n* = 4) days and two (9%) for four or 5 days a week. It was the only weekly outing for 5 participants (22%). Participants had been attending their centres for anything between 6 months and 32 years.

Attenders’ regular activities outside their day centres varied, but most seldom went outside the home. Table 4 provides examples of six attenders’ typical weeks at the time of data collection. Additional File 3 shows examples of two usual weeks.

**Table 4:** Six attenders’ typical weeks

Attender	Typical week
**Bob**	Lives with adult child who frequently travels. Attends day centre twice a week. Goes food shopping twice a week with son or by bus.
**Dorothy**	Lives with adult son. Attends day centre on 3 days. Goes by taxi to church fortnightly with son. Other son visits weekly for an hour.
**Kathleen**	Lives alone in ‘extra care’ (supported living) block of flats. Attends day centre once weekly. Care worker helps with housework for 30 min every morning. Her two adult children each visit weekly (for 4 and 5 h); one delivers shopping and takes her out for a walk. Priest visits to give Holy Communion once weekly (30 min). Hairdresser visits weekly (1 h) and takes to supermarket for food shop fortnightly.
**Rosemary**	Lives alone in ‘extra care’ facility. Attends day centre once weekly. Has regular GP visits on 1 day, after which she does food shopping and attends hospital appointments with voluntary organisation support worker who also does her paperwork (2.5 h) and eats lunch with her at her home. Attends weekly skills centre for visually-impaired to learn computer skills (4 h). Hairdresser visits an hour weekly. At weekends, visits her daughter for one whole day. She attends a monthly two-hour lunch club organised by the day centre provider.
**Thomasina**	Lives alone. Attends day centre once weekly. Adult son visits once a week for 3 h. Cleaner comes weekly for 2 hours, does shopping and sometimes takes her out.
**Wilma**	Lives with adult son and his family; both adults work full-time. Attends day centre once weekly. Attends another day centre once weekly. Privately organised care worker visits every morning (1.5 h), afternoon (30 min) and evening (1.5 h) to change her continence pads, prepare meals and get ready for bed. She also visits on the 5 days that Wilma is not at the day centre to change her pads and make lunch. A cleaner undertakes housework for an hour every week. One daughter visits half a day at the weekend. Another daughter/granddaughter visits for 3 h on the other weekend day. Every month, the local priest visits briefly to administer Holy Communion.

The following paragraphs provide an overview of the whole sample’s contexts.

In addition to their day centre days, the number of days attenders left their homes each week ranged from none to three (average 1.3 days), plus monthly outings. Three-quarters saw family at least once weekly. While two saw family fortnightly or monthly, three saw family irregularly, not at all or had no family. A handful reported at least twice weekly telephone conversations with adult children. Two-thirds had no regular unstructured non-familial social events, such as seeing friends. Those who met with friends relied on friends or family providing transport. Two-thirds had no regular structured non-familial social events. The one-third who did either also attended another day centre, a social club or bingo session weekly, or went on monthly outings or ‘tea parties’ run by the day centre provider or a national charity. Two were enrolled at a skills centre for the visually-impaired. One-third undertook weekly or fortnightly food shopping outings, mostly with support from family, friends, dial-a-ride (mobility service for disabled people) or a voluntary organisation’s support worker. Two attended church regularly, and two received weekly or monthly Holy Communion at home from a visiting priest. One visited a gym weekly.

Two-thirds managed their personal care without paid support, including a handful who had weekly or monthly hairdresser home visits but no other personal care help. Most of the one-third who had personal care received this every day. Just over half of attenders had no paid home care. One-third received once weekly help with housework from home care workers, cleaners or neighbours and, for two, this was daily. Two attenders’ family members undertook housework for them. A handful of attenders had regular medical appointments.

Some participants provided further context about their lives more broadly and their characters. Home maintenance and self-care had become, or was becoming, increasingly effortful for many. While some talked about how independent they had always been, one was still mourning her recent loss of independence. There was a sense of resignation to the situations in which they found themselves, in that some had adjusted to these. A small number mentioned how helpful neighbours were. While some considered themselves to be ‘joiners’ of activities, others said they were not.

## Outcomes of day centre attendance

ASCOT scores and the 10 themes identified from the qualitative and/or quantitative data domains are now presented. Six of the eight ASCOT domains overlapped with the eight themes identified from the qualitative data. Overlaps are covered under the relevant theme headings. Verbatim quotations illustrating commonly expressed or contrasting perspectives (underlining indicates participants’ own emphasis) and ASCOT scores are included where relevant.

The themes are: 1) social participation and companionship, 2) the way time was spent, 3) getting out of the house, 4) improved mental wellbeing and health, 5) practical support, information and access to other services, 6) physical wellbeing and safety, 7) having a meal, 8) accommodation cleanliness and comfort, 9) personal cleanliness and comfort and 10) process outcomes, that is those which pertain to the way services are accessed and delivered.

In qualitative interviews, attenders reported benefiting from attending their day centres. Attendance had added something unique to the lives of all but one attender who later added that the cost was worth it for the change of environment. For one, it had not just ‘added’ something to her life, it had changed it:

‘*It changes your life*.’ (Wilma)

Qualitative outcomes themes (1–7) and process outcomes (10) were reported across socio-demographic and health characteristics, social network types and day centres and across both self-funding and publicly-funded source sub-groups. Themes 1, 4 and 10 were reported by attenders across different marital statuses, ethnicities, age groups, genders, living arrangements, accommodation types, education levels, finances, number of days spent at the centre, number of operational days, EFS frailty levels, PANT network types, groupings (devised for this study) of ASCOT gain (0.00–0.09, 0.10–0.19, 0.20–0.29, 0.30–0.39, 0.40–0.49, 0.50–0.59 and 0.60–0.69) and SWEMWB scores (metric) (15.00–19.99, 20.00–25.99, 26.00–29.99 and 30.00–35.00). Themes 2, 3 and 5 were reported by participants with most of the afore-mentioned characteristics sub-groups, with the following exceptions. Activity-related outcomes were not reported by participants with further education or with highest ASCOT gain score grouping (0.60–0.69). Theme 5 was not reported among married, five-day or attenders with four types of health conditions or disabilities. Getting out of the house was not an outcome reported by attenders with ‘No frailty’, according to the EFS, those whose PANT network was on the borderline between family dependent and locally self-contained, by those aged 70–74, those living with a family member other than spouse or adult child or in private rented accommodation, the single/never-married, those with further education or five-day attenders. Themes 6 and 7 were outcomes gained by fewer than half of the participants, therefore were not represented across all characteristics. Themes 1–7 and 10 were reported by both self-funding and publicly-funded/unsure of funding source sub-groups.

**Fig. 1:**
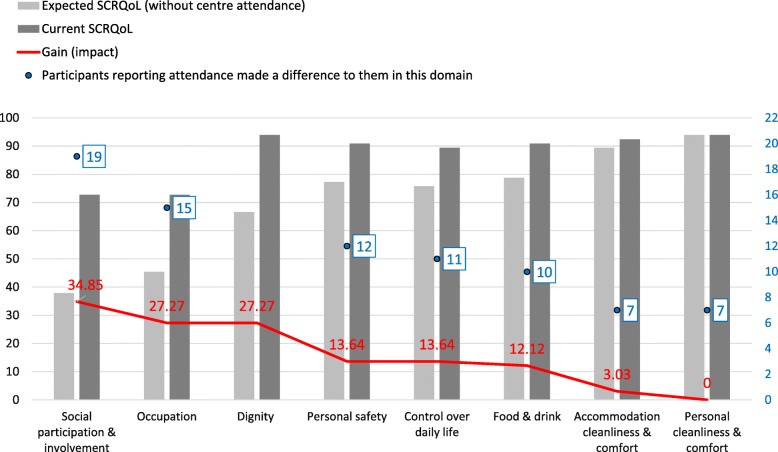
Average unweighted current/expected scores, gain scores and numbers of attenders reporting centres made a difference to them in individual domains

Completion of ASCOT INT4, by 22 attenders, also indicated that quality of life improvements were directly attributable to day centre attendance, covering themes 1, 2, 4, 6, 7, 8 and 9. Figure 1 shows average ASCOT current and expected SCRQoL score in each domain as a percentage of the total possible score (unweighted), gain in each domain and numbers of attenders saying that centres made a difference to their lives in each domain). It shows that the difference between the with and without scores (of a maximum of 100) was 34.85 for social participation and involvement and 27.27 for occupation; that is almost one-third higher with than without the service. These impact scores are statistically significant (chi-squared test).

Overall average preference-weighted SCRQoL scores were 0.88 (current) and 0.70 (expected), with a resulting overall quality of life gain of 0.18 resulting from day centre attendance. The gain of 0.18 is a preference-weighted score (gain can be − 0.17 to 1 where 1 is the ideal situation). Thus 0.18 is not statistically significant but is an indication. Average gain varied between centres (0.13, 0.15, 0.16, 0.24) and between individuals (individual gain score range 0.00–0.62). Gain scores may have undervalued attendance’s actual impact on some participants’ quality of life. For instance, a small number of attenders’ answers did not always correspond with their qualitative interviews, and clarification of questions’ meaning was needed in some cases. Furthermore, participants reported attendance making a difference to their lives when responding to ASCOT questions even when gain scores were low or nil. Notwithstanding socio-demographic and health variations between centres, in three centres, overall expected SCRQoL, in the absence of day centre attendance, was 0.69 and 0.75 in the fourth. Average gain was higher for publicly-funded attenders (0.24) than for those self-funding (0.15).

### Theme 1: day centre attendance provided access to social participation and companionship

‘Social participation and involvement’ was one of two ASCOT domains in which centre attendance made a significant impact (*p*-value < 0.001, 99% CI) on participants as a group. Centre attendance was said to address the problem of not meeting people when physically unable to get out of one’s home. Companionship was one of the unique contributions that participants reported centre attendance made to their lives. There were different facets to this most talked-about outcome.

Many valued having social contact which contrasted with usually being alone:

*‘It’s like, if somebody is married and they are not happy in their marriage, they look for a way out. Well I am not happy being at home on my own and so that’s my way out.’* (Tina).

*‘I get conversation instead of talking to myself… And I’m mixing with human beings.’ (*Nellie*).*

Attenders enjoyed doing things in company:

*‘We sit together and play together, like cards or any other games or … the memory class, and of course, the exercise.’* (Mariana).

The group environment enabled the opportunity for laughter or fun. There was a good deal of banter in some centres, sometimes group-based, or one-to-one between attenders or one-to-one with staff or volunteers:

‘*I think that’s one of the things that I like about it. You have heard [female attender] and I****roar****with laughter before now, haven’t you? …I can make her laugh so easily. I love it. I know when she laughed her head off 1 day when one of the questions was what did Richard II lose in the bushes and I called out, “his virginity.” She said, “for God’s sake.” I don’t know. I just like laughing anyhow*.’ (Kaye  - emphasis underlined and emboldened).

Participants highlighted how they looked forward to regular contact with day centre friends:

‘*You look forward to seeing friends again, you know*.’ (Elizabeth).

There was mostly no contact between attenders outside centres on non-attendance days; some considered it unnecessary as they saw each other regularly at centres. For others, co-attenders were simply acquaintances whom they saw regularly at centres:

‘*They are just Friday people*.‘ (Thomasina)

Around half of the participants mentioned that increasing proportions of cognitively impaired attenders, either due to dementia or a learning disability, impacted negatively on levels and quality of connection possible. Although one attender referred to a co-attender living with dementia as ‘*a sweet little thing*’ (Jenny) and was impressed how well she joined in with games, she considered that the latter’s poor memory had hindered the development of friendship. Another reported that she was not able to build a relationship with some of her co-attenders was because ‘*you are talking to them and they are just looking at you*’ (Olive).

A small number also commented upon conversational *faux pas* and what they described as ‘annoying’ or disruptive behaviours, such as constant rocking, swearing or making unintelligible burbling noises which ‘*would be frightening to some people*’ (Francine) or showing aggression:

‘*Some are a little bit annoying (…) One time she started smacking people with her stick*’ (Elizabeth)

Two of the five male participants expressed a preference for higher numbers of men since they maintained that men and women chat about different things.

### Theme 2: day centres provided something (different) to do

The second ASCOT domain in which centre attendance made a significant impact (*p*-value < 0.001, 99% CI) on participants as a group was that of ‘occupation’, or meaningful activity. This was also a unique contribution centre attendance made to participants’ lives. Attendance was an activity which meant doing something instead of doing nothing at home:

‘…*just quite different to what you are doing at home’* (Kaye).

*‘I just sit here from when I get up to the time I go to bed.’* (Ruby).

Having regular day centre days gave some attenders purpose within their week:

‘…*something to wake up in the morning to do*’ (Nellie).

It also gave them something to think about, something also benefiting family relationships:

‘*It’s enriched my life (…) Well I suppose it gives me an interest, doesn’t it? It’s a big interest. And it gives me something other to talk about and to think about.’* (Jenny).

Attenders enjoyed being occupied or they enjoyed specific activities they did at their centres. In most centres, activities were varied although not all attenders’ (mainly sensory impairment) needs were always catered for. Joining in these was said to be enjoyable, stimulating, and, in some cases, satisfying:

‘*I like to be doing something.’* (Lenny).

**‘***I****enjoy****the art. I****enjoy****the singing. I****love****to sing. I don’t mind whether there is one or two singing.’* (Wilma).

Activities cited as particularly enjoyable were art, craft, cooking, computer classes, charades, discussion groups, memory exercises, raffle/tombola, singing and music sessions, sweet shop, trips out, poetry reading, food tasting, table games, listening to background music, reading the paper, sitting in the garden, visiting speakers, therapy dog visits and performances by folk and belly dancers. Quizzes, bingo, card games, and exercise provoked mixed reactions.

### Theme 3: day centres provided the opportunity to go out and have a change of environment

A qualitative theme, being enabled to ‘get out of’ their home, another of centre attendance’s unique contributions to their attenders’ lives, was framed in two ways.

Firstly**,** it was tantamount to escaping from their home in which they felt they were ‘stuck’, or even imprisoned:

‘*It’s like being a prisoner in my house now … That’s how it feels now and again, because you don’t****seen****obody there now*.’ (Olive).

Others did not feel imprisoned and were more concerned with having a change of scene:

*‘Well, it gets me out of my four walls for a start.’* (Nellie).

*‘Well, it gets me****out****once a week, which I wouldn’t do otherwise.’* (Ruby).

Secondly, the day centre was somewhere to go when you had ‘*nowhere else to go*’ (Nellie). It was also a place to go and gather; saying hello to an acquaintance in passing was ‘*not the same as actually going to a function with the people*’ (Bob).

There was much stoicism and acceptance of current situations in which some attenders said they would never have imagined themselves. For some, centres appeared to be a good substitute for what they may have preferred to do had their abilities been different:

*‘I used to like going here, there and everywhere. Now I can’t****do****that so I don’t mind coming here…I am happy with it…It gives me a chance to come out.* (…) *if I could go round and do things that I would like to do and so I’d go shopping and maybe walk around.’* (Dorothy).

### Theme 4: improved mental wellbeing and health

Participants reported improved mental wellbeing and health as a further unique contribution of attending their centres. Not only did participants enjoy certain aspects of what was provided by centres, but many enjoyed the whole experience.

**‘***Oh, I****love****going. Oh yes. Yes.*’ (Kenneth).

‘*Well it’s my life. It’s all I’ve got. It literally****is****my life*.’ (Nellie).

They gained a sense of purpose, felt like they belonged, felt in control or more independent and ‘felt better’ generally. The ASCOT domain of control, the third highest scoring, falls within this theme.

They had something to look forward to that they enjoyed – and some found fun. Enjoyment was derived from social contact, the activities, getting out of the house, feelings of freedom, the meals and additional extras linked with a centre’s location, transport to it and to attending a centre as an activity in itself. Attendance also counteracted boredom and life’s monotony, helped participants gain a better perspective of their own situations and feel more relaxed, less lonely or depressed or more confident, mentally stimulated or energised. Centres were also referred to as a ‘lifeline’.

*‘I just enjoy it there. Because I am alone. I am on my****own****. Sometimes I feel****sad****. I feel better when I go to the centre I have a little bit of talking, conversation and some socialising. (…) I enjoy it very much. To tell you the truth, before Monday I had been waiting for Monday to come. (…) I feel happy and it helps my depression.’* (Miguel)

Furthermore, participants felt valued and respected as individuals which reflected centre staff’s and volunteers’ personalities, behaviour and their delivery of the service (see Theme 10). The ASCOT domain of dignity, the second highest scoring, falls within this theme.

### Theme 5: practical support, information and access to other services

A qualitative theme, practical support, information and access to other services were either provided as part of the day centre service, via occasional or regular visiting professionals, speakers or other centre visitors, or were other services offered by the day centre providers. Most mentioned were the supply of hearing aid batteries or maintenance, and useful talks. Other examples provided were lunch clubs, holidays, shopping trips, hairdressing, fingernail filing and painting, help with arranging health or other appointments, referrals to, for example, occupational therapy to get a shower installed at home, or to the local authority to get a personal falls alarm installed, help to claim taxi vouchers (discounted taxi fares) and visiting chiropodists or massage therapists or clothes-sellers. Benefits gained included, for instance, feeling safer or having peace of mind, enjoying trips out, saving or having more money:

*‘Before that I was buying the batteries because… I could get them free from the hospital but I’d have to take a taxi to the hospital to get them. So I used to buy them from [pharmacist]. But [day centre manager] said “oh no, don’t buy them. We’ll give them to you.” … That’s another thing that’s been a great help. (…) I can clean part of it but I can’t take the things apart and clean it properly.’* (Francine).

Not only did Ruth feel more settled at her day centre after a group holiday with other attenders, which she accessed through the centre, but it also helped reduce her depression:

*‘And then eventually heard about the holiday (…) I****made****myself go and it did me the world of good because since then, my****dark****side seems to have lifted. Although I’ve physically got all these problems, mentally I’m fine now, really.’* (Ruth).

Co-located facilities were a bonus. At one centre, the short, midweek religious service was attended by some participants. At another, the library was appreciated by a keen reader who also enjoyed occasional contact with babies at the mother and baby group, as was an advice service.

### Theme 6: physical wellbeing, health and safety

This theme comprised three parts.

First, attenders reported benefiting from informal health and wellbeing monitoring and follow-up undertaken by day centre personnel, such as being asked how they were or if something was the matter, which they appreciated. Staff, and volunteers, listened to attenders talk about continence or pain, for example, and, with attenders’ consent, spoke to named relatives about health concerns:

‘They come around asking “*Are you alright? What’s the matter?”’* (Thomasina).

They also measured blood pressure, made GP appointments, reported safeguarding matters (e.g. about possible elder abuse) to the local authority and replaced a screw in one attender’s reading glasses.

Second, exercise was felt to help maintain mobility and alleviate depression:

*Interviewer: ‘So what is it about being here that helps you feel less depressed?’ Denzel: ‘Well, I do exercise three times a week.*’

Attenders felt they were more likely to exercise in a group than alone at home:

*‘I think I quite enjoy it when there is people come and give us exercises and things, you know, make us to do things. (…) I think it’s good for us. (…) I can sit here all day and not move. [Laughs] I could move, but I think it’s good to make you do a few exercises. If you’re all doing it, you do it.’* (Ruby).

Third, some attenders felt physically safer and less vulnerable at centres compared with at home. ASCOT’s third highest scoring domain of gain, personal safety, although broader in scope, falls within this sub-theme. One attender spoke about feeling vulnerable at home after a doorstep incident with rogue traders, and another, suffering from vertigo, said:

*‘Well, I come to the club when I not in hospital. You feel more safer. If I here and anything happened to me, they will call the ambulance.’* (Norma).

### Theme 7: having a meal (food & drink)

Although a minor outcome theme arising from qualitative findings, almost half the participants said that day centre attendance made a difference to them in the ASCOT domain of food and drink, and most shared their, mainly positive, views of the meals provided. Reasons for reporting having a meal as an outcome included being unable to stand for long periods when cooking, closure of a lunch club, wanting ‘*a meal put down in front of me without having to cook it myself’* (Isobel). The opportunity for conversation over lunch was welcomed. Negative comments, concerning one centre only, included long waiting time, lukewarm food, feeling rushed, poor variety and disliking the meals. Meals are categorised separately from physical wellbeing and health since people would be eating lunch at home on non-day centre days.

### Theme 8: accommodation cleanliness and comfort

A theme emerging from quantitative data only, centre attendance was reported to make a difference to just under one-third of participants in this ASCOT domain, but the average gain score was very small.

### Theme 9: personal cleanliness and comfort

Another theme emerging from quantitative data only, centre attendance was reported to make a difference to just under one-third of participants’ quality of life in the personal cleanliness and comfort ASCOT domain, but average gain was zero. However, while responding to the tool’s questions, two participants implied that attendance did impact positively on them: one commented that attendance affected how clean he felt since he bathed and wore his best shirt on attendance days and the other said she took care of her appearance as she knew men would be present.

Two of the day centres had bathing facilities (suitable for people with disabilities), but managers reported attenders using these only in emergencies.

### Theme 10: process outcomes

While certain experiences contribute to the overall centre attending ‘experience’ (e.g. activities offered), others contribute towards ‘process outcomes’. Examples include feeling valued and respected, being treated as an individual, having a degree of control over the way a service is delivered, the extent to which a service fits with other support received and value for money [54]. Some of these outcomes (identified from both datasets) have already been alluded to, particularly under Theme 4, Improved mental wellbeing and health.

Overall, reported experiences and feelings about day centres indicated that attenders experienced mainly positive process outcomes. All planned to continue attending and would recommend their centre to friends, family or somebody in the same situation as themselves. Many considered their centre offered good value for money.

*‘Yes, its good value for money*.’ (Miguel)

Although a very small number of attenders reported mixed feelings on centre attendance mornings and making themselves get ready as they knew they would enjoy it once there, feelings of positivity were widespread, with many enjoying the whole experience, looking forward to or loving it.

‘*I think it’s the****best****thing they have done, [local authority], make this place (…). they do a wonderful job here. I don’t think I’d rather be anywhere else but here. I really****do****enjoy being here (…) I am glad I come.’* (Isobel).

‘*All I can say is that, anyone who doesn’t go there is missing out on something. I like it there and I think it’s wonderful.*’ (Kathleen).

Comments on centre personnel were overwhelmingly positive. The few criticisms related to attenders exhibiting unpleasant or disruptive behaviour not being dealt with, certain staff very occasionally being a bit domineering or lacking understanding of sight loss. In one centre, three attenders but were less enthusiastic about one staff member than others but did not ‘dislike’ her.

The ASCOT domains of dignity (personal sense of significance) and control, the second and third highest scoring, fall within this theme. It is likely that less positive feelings, a judgement that attendance may not have been such good value for money and, perhaps, fewer attenders planning to continue attending may be been more apparent had attenders not found themselves feeling valued, respected, treated as an individual or with a degree of control over service delivery (see Theme 4):

‘*The staff of [day centre] are the salt of the earth. (…) They are always there to****help****. No matter what, they are always there, ready. Nothing to fault them for. They are very ordinary, very friendly and they are****wonderful***.’ (Tina)

## Discussion

### Principal findings

This study provides new understandings of the circumstances behind people’s motivations to attend a day centre and of attenders’ lives, going beyond their socio-demographic details, and how they accessed centres. This paper reports individual contexts, in the form of short narratives and grouped experiences, which were previously largely unexplored, and which are useful for offering a richer context in which to situate attenders’ outcomes. Participants exhibited advanced age, declining health and mobility, sensory loss, bereavement and were retired, all of which are risk factors for social isolation [55], and their social network types put many at risk of mental ill-health [44]. Attenders’ profile places them within the National Institute for Health and Care Excellence’s (NICE) category of ‘vulnerable older people’ who are ‘*most at risk of a decline in their independence and mental wellbeing*’.(56:para 1.5.3). There was low awareness of the existence of day centres prior to attending one and acess to day centres had mostly been facilitated for attenders by social care or health professionals or family, but few by GPs.

This study also provides new understandings of what are ‘the outcomes that matter’ for cognitively intact older people attending day centres. Attendance enhanced quality of life, sometimes significantly, and made a unique contribution to their ‘vulnerable’ attenders’ lives [56] thus demonstrating their policy-relevance.

Day centres emerged as life-enriching gateways to companionship, activities, the outside world, practical support, information, other services, the community and enjoyment for people who had experienced loss, were socially isolated and unable to go out without support. They appear to promote elements of successful ageing, by enabling attendees to maintain social connections, compensate for mobility problems and social isolation, and optimising opportunities for company and chosen activities [57]. A major contribution to participants’ mental wellbeing was through centres supplying a source of enjoyment or fun which significantly contributes to wellbeing [58] with laughter having a positive psychophysiological impact [59].

Centres were communities of choice that ‘enabled’ and offset loss or isolation, in the absence of other suitable options, and community resources that supported people in adverse circumstances, thus supporting ageing in place through wellbeing*.*

People growing up after World War II are said to recognise welfare as a reassuring ‘safety-net’ (i.e. ref [60], page 59). Day centres were identified as providing attenders with two such ‘safety-net’ outcomes: monitoring attenders’ health and wellbeing, and providing practical support, information and facilitating access to other services. Thus, centres offered added value beyond the purposes for which they were commissioned or funded, beyond what may be assumed to be covered by a service aim of improving quality of life or supporting people to remain at home, and beyond what attenders may have wanted or expected, given their reasons for attending. These added value outcomes emphasise centres’ underlying nature of being long-term maintenance and monitoring services rather than services that deliver specified improvements after which people are discharged. Importance has been placed on single entry points to minimise people ‘falling through’ gaps between services [61].

Notably, outcomes were achieved despite three-quarters of participating attenders using centres for only one or 2 days a week (i.e. 4.5–12 h excluding travelling time), something perhaps attributable to the fact that many outcomes fall within the higher order categories of human need [62].

### Comparison with other work

Attenders’ characteristics cannot be benchmarked against an existing dataset due to the lack of national data about English day centres. This study found attenders were people whose health and mobility had already declined; this decline was due to loss (e.g. of good health) or had resulted in loss (e.g. of social contact). This profile contrasts sharply with that of attenders in studies undertaken in the 1970s–80s, more of whom was younger and more active [63, 64]; during these decades, publicly-funded social care eligibility criteria covered people with low and moderate needs and more ‘low-level’ day centres operated [12, 65]. In the 2000s, increasing disability among English day centre and lunch club users was noted [66] and, in the 2005–17 literature, centres were reported to be mainly used by people whose health had begun to decline [11]. Further corroborating this finding was the exclusion from the present study of 41% of observed attenders due to dementia-related cognitive limitations. This proportion is double the 19% of publicly-funded day centre attenders with dementia reported in the 2013–14 annual survey of Councils with Adult Social Services Responsibilities [4]. This is also despite this present study including older people not referred by a LA or being funded to do so, and some participants reportedly having a diagnosis of dementia. This present study also indicates that generalist day centres – those without the identity of specific nationality or ethnicity-focussed provision – may be acceptable to people from black and minority ethnic backgrounds.

Day centres remain a gendered service. The literature has persistently failed to note the over-representation of women among attenders [67]. In this study, women accounted for three-quarters of participants (and attenders overall). Although women account for 60% of people in England aged 80 and older and 55% of people aged 68 and older [youngest study participant’s age] [68], morbidity rates are higher among older women than men. Also remaining unchanged is their attendance by widowed/single people and people living alone. Feeling the need to get out of the house for a change of environment was a motivation for attendance not identified in the recent literature [11].

This study reports attenders’ religious affiliation, sexual orientation and gender reassignment, three of the nine protected characteristics stated in the UK Equality Act 2010 which cannot be used to treat people unfairly (also age, marriage/civil partnership, disability, race, sex, and pregnancy or maternity) [69], characteristics that were, hitherto, absent from the literature [11, 67]. Such data are needed to enable day centres to consider matters concerning access to centres and to respond to the challenges of supporting different user groups [67].

Social participation related improvement was more widespread compared with the only other identified day centres study employing ASCOT [13], perhaps reflecting this present study’s older and less mobile participant profile and because the strongest effects of social activities are felt amongst those least physically active [70]. Day centres have not previously been conceived as gateways, as sources of enjoyment, or as offering added value. Only day centres in one Bahranian study aimed to provide ‘fun’ [71]. People have been reported to enjoy attending centres and to laugh there, but this has previously been conceived as contributing to overall life satisfaction [72, 73] rather than enjoyment being considered as an outcome in its own right. Enjoyment of activities has been conceived as contributing to whether attending has made a difference to people’s lives [74]. Findings confirmed two previous English studies reporting day centres’ high emphasis on process outcomes [75] and attenders’ high satisfaction with their relationships with day centre workers, workers’ behaviour and their work [13].

As part of the Adult Social Care Outcomes Framework (ASCOF), the local authority Adult Social Care Survey (ASCS) gathers quality of life data using a different eight-question version of ASCOT. Current ASCOT INT4 quality of life scores are comparable with this data if agreggate, non-preference-weighted scores are calculated. It should be noted, however, that publicly-available ASCOF data are not separated by service type. Covering part of the same period, the 2016–17 ASCOF in England [76] reported an average current SCRQoL score of 18.9 across England among service-using respondents aged 65 and older; the average across the four local authorities participating in the present study was 18.95. Study participants’ average score of 19.4 compares favourably to these figures when converted to be comparable [38].

The contextual data about day centre attenders’ lives beyond the centres are missing from the literature and are a unique contribution of this study, thus expanding the evidence base on which to make clinical commissioning decisions [15].

### Meaning of the study and implications for clinicians and policymakers

In line with Outcomes Frameworks, this study identified mainly positive experiences and evidence that outcomes of day centre attendance address those targeted by health and social care policy. Centres supported their mainly socially isolated and housebound attenders to age in place by focusing on their wellbeing and by preventing deterioration, and acted on any safeguarding or health concerns. Since centre attendance was linked with non-elective withdrawal from social participation, for various reasons, and centres facilitated valued social contact, activities and interventions that improved quality of life, there is potential for day centres to feature on social, or wellbeing prescriptions. Social prescriptions are non-medical interventions whereby primary care professionals refer people with social, emotional or practical needs to non-clinical services with a goal of promoting improved outcomes [77].

Outcomes are of consequence due to the rising numbers of older people living alone [78] and because age influences social exclusion, with people aged 85 or older at greatest risk [79]. Getting out is something older people with high support needs want to do and value [80]. Feeling ‘stuck’ at home (i.e. ref [81], page 59) due to mobility restrictions may lead to social isolation which has a negative health impact [26]. Very old people (≥85 years) spend an average of 80% of their time at home [24]. Staying at home for most of the time due to health constraints is significantly associated with poor wellbeing [25]. Promoting positive wellbeing makes a real impact on older people’s lives and contributes to reducing future demands on health and social care services, families and communities [82]; attenders with a similar profile to this study’s participants may have needed publicly-funded care and support if their centre ceased operating.

### Strengths and weaknesses of the study

The study’s strengths lie in its focus on generalist day centres, its in-depth nature, and its focus on entire experiences of and outcomes of day centre attendance as one single intervention rather than of specific interventions in centres or specific aspects of experiences. Combining a quantitative measure with qualitative interviews added richness and depth, enabling quantification of some of the outcomes emerging using both methods, and providing insight into factors contributing to outcomes experienced and the significance of these to attenders. As a cross-sectional study, the findings provide an in-depth snapshot of 1 day at each centre rather than a longitudinal overview. However, use of an ASCOT tool that measured of hypothetical outcomes in the absence of service at a single time point aimed to compensate for this to a degree. Valuable qualitative data, captured in field notes, also arose while administering a validated tool (see Theme 9), suggesting that following Abendestern et al.’s [83] novel approach of recording conversations during completion of validated tools may broaden and strengthen findings. Data collected using validated scales are comparable. Rigour was maximised by lay scrutiny of interview questions, undertaking regular visits which habituated participants to the researcher and led to a trusting rapport, recording and transcription of interviews and taking a systematic approach throughout. The interviewer kept a reflective diary. Findings’ transparency and trustworthiness are reinforced by feedback from the Study Advisory Group and representatives from each case study site. Their validity is enhanced by the diversity of participating centres despite these not reflecting all typologies. Centre diversity and the emergence of common themes across centres compensated for its limitations which relate to the low number of participating centres in one English region and small sample sizes. Frailer and less cognitively able attenders may be under-represented, although participation was high among those eligible, and, in conversation during fieldwork, many non-participants expressed similar views and experiences to the participants. A risk of bias is that poor-quality day centres may not have agreed to participate.

## Conclusion

Generalist, traditional day centres are no longer the ‘low-level’ services they once were. While centre attenders share some characteristics, they are not homogeneous, although many are socially isolated, due to their circumstances, and are unable to go out without support. Loss and a resulting desire for something different in their lives were driving factors for attendance. Day centre access was mostly facilitated for older people due to their lack of awareness of the existence of day centres. As resources to address the risks of loneliness and isolation [26], these are promising interventions that are acceptable to some and accessible. By supplementing or replacing inadequate or insufficient informal care, support, social networks or other opportunities for vulnerable people who are housebound but not necessarily frail, centre attendance counterbalances some of the potentially negative consequences of ageing in place with mobility restrictions and improves quality of life. This suggests the value of promoting professionals’ ability to recommend consideration of day centre attendance in the context of increased interest in social prescribing [6]. This research highlights the value and complementarity of mixing qualitative and quantitative methods.

With policy encouragement of integrating health and social care commissioning in England, further research could usefully explore NHS professionals’ and commissioners’ awareness of and views on generalist day centres, their purpose and potential, for example about centres’ health and wellbeing monitoring role. Further research might also explore the costs of day centres and any expenditure-related benefits linked with their use, for example the relationship between health and wellbeing monitoring at day centres and the use of primary or secondary care.

## Supplementary information

**Additional file 1.** Older attenders of day centres: qualitative semi-structured interview guide

**Additional file 2.** Coding frame: Attenders’ circumstances, motivations and outcomes

**Additional file 3.** Illustrative ‘Maps of a Usual Week’

**Additional file 4.** Summary of data informing outcomes themes

## Data Availability

All data generated or analysed during this study and relevant to this paper are included in this published article. The data are not publicly available. This manuscript is an honest, accurate, and transparent account of the study being reported; no important aspects of the study have been omitted. See Additional File 4 for a summary of data informing outcomes themes.

## Abbreviations

ASCOF: Adult Social Care Outcomes Framework
ASCS: Adult Social Care Survey
ASCOT INT4: Adult Social Care Outcomes Toolkit, Version INT4
DCHA: Participating Day Centre - Housing Association
DCLA: Participating Day Centre - Local Authority
DCV1: Participating Day Centre - Voluntary Sector (1)
DCV2: Participating Day Centre - Voluntary Sector (2)
PANT: Practitioner Assessment of Network Type
SCRQoL: Social care-related quality of life
SWEMWBS: Short Warwick-Edinburgh Mental Wellbeing Scale
TUG: Timed Up and Go test
