# Supplementary material for: Day centres for older people - attender characteristics, access routes and outcomes of regular attendance: findings of exploratory mixed methods case study research
Source: BMC Geriatr. 2020 May 4;20:158. doi: 10.1186/s12877-020-01529-4 (PMC7197165; doi:10.1186/s12877-020-01529-4)
Supplement: Supplementary file 1 — Additional file 1. Older attenders of day centres: qualitative semi-structured interview guide [file 12877_2020_1529_MOESM1_ESM.pdf]

ADDITIONAL FILE 1

Older attenders of day centres: qualitative semi-structured interview guide

Contextual information – what a usual week looks like

We'll start by thinking about what a usual week looks like for <u>you</u>. Using an A3 print-out of a 'map of a usual week' divided into days of the week and morning, afternoon and evening, interviewer guides conversation about what happens during what interviewees consider to be a 'usual' week for them and fill in the 'map'. Anything fortnightly/monthly will be marked as such. <u>Aim</u>: to cover all informal and formal support and activities: any care/support they receive (family, relatives, friends) or which they pay for [who from? how paid?], any activities they attend or are involved in, social/other visits received, outings etc.

Why person attends, their experiences, outcomes and connections with other attenders outside the day centre

Can you tell me how you travel here?

Would you like to tell me what started you thinking about coming to a day centre like *[name of day centre]*?

What did you think of day centres such as [*name of day centre]* some time ago, before coming here?

Have you made any friends at [*name of day centre*] who you see, or are in touch with by phone, outside the day centre?

On a practical note, have you been given any information, got involved in other activities or used other services through *[name of day centre]*?

What, if anything, has changed for you as a result [of these]?

Can you tell me about how you've found the whole experience of coming here?

How do you pay for this day centre service? [If you don't pay: who pays the costs of the centre?]

If you were unhappy with anything, would you know what to do about it and would you feel comfortable bringing it up?

Would you recommend coming to [*name of day centre*] to friends, family or a newcomer to the area who is in a similar situation to yourself?

Would you say that coming here adds anything to your life?

Please could you describe the two things you like best about coming to *[name of day centre]*?

Please could you describe the two things you like least about coming to *[name of day centre]*?

Do you plan to continue coming here? If not, why is that?

If you have a relative caring for you, do you think that you coming to [*name of day centre*] helps them in any way?

Is there anything else you'd like to tell me about [name of day centre]?

Authors: Katharine Orellana, Jill Manthorpe, Anthea Tinker
