# Supplementary material for: Day centres for older people - attender characteristics, access routes and outcomes of regular attendance: findings of exploratory mixed methods case study research
Source: BMC Geriatr. 2020 May 4;20:158. doi: 10.1186/s12877-020-01529-4 (PMC7197165; doi:10.1186/s12877-020-01529-4)
Supplement: Supplementary file 2 — Additional file 2. Coding frame: Attenders’ circumstances, motivations and outcomes [file 12877_2020_1529_MOESM2_ESM.pdf]

## ADDITIONAL FILE 2

## Coding frame: Attenders' circumstances, motivations and outcomes

| THEMES | CIRCUMSTANCES WHEN STARTING TO THINK ABOUT DAY CENTRE ATTENDANCE | PRIMARY MOTIVATIONS FOR DAY CENTRE ATTENDANCE | OUTCOMES (*unique contribution) |
|---|---|---|---|
| **ACTIVITY-RELATED** | Closure of other DC or club / other DC criteria changed<br>Had to stop volunteering<br>Retirement<br>Something to do<br>Somewhere to go<br>To keep mind alive | Something helpful to do after retirement<br>To keep mind alive<br>Wanting to do activities/something | Activities keep mind occupied/stimulated<br>DC attendance keeps your time occupied<br>Different to what would be doing at home<br>Doing something vs nothing at home<br>Done things wouldn't have done otherwise (e.g. craft, trips out*)<br>Given something to do/think about -an interest-conversation material<br>Specific activities (went there for) |
| **CARER-RELATED** | Carer needed a break<br>Isolated as a spousal carer<br>Attended with husband who cared for (unclear why) | Attended with husband who cared for (unclear why) | |
| **CHANGE OF ENVIRONMENT/ GETTING OUT OF HOUSE** | Not getting out enough<br>Stuck at home | Want to get out / a change of environment | Access to a garden<br>Gets out of house/4 walls (prison/boring)<br>Nowhere else to go-only way to get out-gives chance to go out*<br>Place to go without difficulties of trips out* |
| **LOSS OF MOBILITY (independence)-physical health** | Couldn't get out without help-transport<br>Decline in physical health – sudden<br>General decline in physical health<br>Loss of car-stopped driving (independence) | | |
| **FOR MEALS** | | For meals | Having a meal |
| **MENTAL WELLBEING/HEALTH** | Anxiety disorder<br>Depression -  felt low/down<br>Felt lonely<br>Lacked confidence | To address diagnosed mental illness | Boredom-monotony broken-change-break<br>Changed-enriched life-opened up new life*<br>Enjoyment-fun-laughter*<br>Feel more stimulated mentally<br>Feels energised - motivated |

**Day centres for older people: attender characteristics, access routes and outcomes of regular attendance. Findings of exploratory mixed methods case study research.**

Authors: Katharine Orellana, Jill Manthorpe, Anthea Tinker

| | | | | |
|---|---|---|---|---|
| | | | Gained perspective of own situation<br>Lifeline-lifesaver<br>Other - own attitude matters<br>Feeling part of a group-belonging*<br>Sense of independence & control*<br>Sense of purpose - feeling useful to others-making a contribution-active role to play-opportunity to do so*<br>Sense of purpose/structure within own life<br>Feelings of freedom*<br>Nothing added to life | |
| | | | Feeling better* | Less lonely*<br>Less depressed-sad/makes feel happy*<br>More confident & relaxed*<br>Helped to keep sanity* |
| **PHYSICAL HEALTH/ WELLBEING/SAFETY** | | Exercise<br>Exercise - referred to drop-in GROUP rehab exercise (health) | Do more exercise<br>Health monitoring (& outlet for problems)<br>Safe place-feeling safer | |
| **PRACTICAL SUPPORT, INFORMATION & OTHER SERVICES ACCESSED** | | | What accessed | Other services used (DC provider)<br>Personal alarm system<br>Services of another provider<br>Taxi vouchers-card |
| | | | Within DC | Chiropodist<br>Clothes<br>District nurse now treats at DC<br>Hairdresser<br>Hearing aid maintenance-batteries<br>Help with appointments (staff-vols)<br>Information & useful talks<br>Massage<br>Nails filed & painted (staff-vols)<br>Staff-vols helped access something |
| | | | Made a difference | More aware of certain things<br>More money |

| | | | | |
|---|---|---|---|---|
| | | | | Safer - peace of mind<br>Saved money<br>Saved trips elsewhere<br>Did not say whether made a difference |
| **SOCIAL INTERACTION / COMPANIONSHIP** | Alone (not bereaved)<br>Bereavement (spouse-partner)-was on own<br>Insufficient contact with peers<br>Insufficient contact with people – socialising<br>Loss of existing social networks (non-group)<br>Loss of outside activities - stopped attending other group/club<br>Attender naturally sociable/joiner | Needed-wanted to socialise-see people<br>Contact with peers | Companionship*<br>Company makes a change<br>Eating in company<br>Friends - made new ones<br>Friends - reconnected with old ones<br>Laugh & fun & joke*<br>Conversation material<br>Proper conversation about things that really matter* | |
| | | | Contact with people | Own age group*<br>People generally<br>Arguing<br>Chatting<br>Hearing people<br>Seeing people<br>People who are also lonely-similar circumstances<br>A range of different people |
