# Supplementary material for: Day centres for older people - attender characteristics, access routes and outcomes of regular attendance: findings of exploratory mixed methods case study research
Source: BMC Geriatr. 2020 May 4;20:158. doi: 10.1186/s12877-020-01529-4 (PMC7197165; doi:10.1186/s12877-020-01529-4)
Supplement: Supplementary file 3 — Additional file 3. Illustrative ‘Maps of a Usual Week’ [file 12877_2020_1529_MOESM3_ESM.pdf]

ADDITIONAL FILE 3

Illustrative ‘Maps of a Usual Week’

ACTIVITY TYPES:
- MEDICAL
- SOCIAL
- EDUCATIONAL
- FAMILY
- PAID CARE
- DAY CENTRE
- OTHER

Nellie

	Hour	Monday	Tuesday	Wednesday	Thursday	Friday	Saturday	Sunday
	7							
Morning	8	Keeps whole day free for: - blood tests						
	9	- delivery of medication; Cleaner hoovers	Weekly trip to Tesco. Has permanent booking with Dial-a-Ride for transport.				At home all day. Does laundry.	
	10			10.30-3 DAY CENTRE Self-pay	*MONTHLY* *Coffee mornings at volunteeers' homes (organised by day centre provider) (Transport with volunteer driver)*			
	11					*MONTHLY* *Lunch club organised by day centre provider. (Transport with volunteer driver)*		
	12							
Afternoon	13							
	14							*MONTHLY* *Contact the Elderly tea party in a volunteer's home.*
	15							
	16							
	17							
Evening	18							
	19							
	20							
	21							

Elizabeth

	Hour	Monday	Tuesday	Wednesday	Thursday	Friday	Saturday	Sunday
	7							
Morning	8							
	9				Daughter visits			
	10		10 Volunteer-driven car; 10-3 DAY CENTRE Self-pay				Local friend visits (Saturday or Friday)	
	11				Sister & brother-in law visit (11-12.15)			
	12							
Afternoon	13							
	14							
	15		Volunteer-driven car (3-3.15)					
	16							
	17							
Evening	18							
	19		Bingo with neighbour (7.30/8 - 9.30/9.45). Neighbour drives.					
	20							
	21							
