# Supplementary material for: Day centres for older people - attender characteristics, access routes and outcomes of regular attendance: findings of exploratory mixed methods case study research
Source: BMC Geriatr. 2020 May 4;20:158. doi: 10.1186/s12877-020-01529-4 (PMC7197165; doi:10.1186/s12877-020-01529-4)
Supplement: Supplementary file 4 — Additional file 4. Summary of data informing outcomes themes [file 12877_2020_1529_MOESM4_ESM.pdf]

ADDITIONAL FILE 4: Summary of data informing outcomes themes

Outcomes themes	QUALITATIVE DATA (n=23)			QUANTITATIVE DATA (ASCOT INT4) (n=22)	
	Attenders reporting this outcome	Attenders reporting this unique contribution to their lives	Total no. of references within theme	Attenders responding that attendance made a difference to their lives in this domain	SCRQoL gain
**Social participation/ companionship**	21	11	247	19	Highest gain
**Activities/changing way spent time**	17	6	117	15 (occupation)	Joint second highest gain
**Mental wellbeing & health**	20:<br>- enjoyment (n=18), laughter and fun (n=7)<br>- sense of purpose (n=5)<br>- sense of independence, freedom and control (n=3)<br>- boredom/monotony kept at bay (n=5)<br>- gained better perspective of own situation (n=4)<br>- felt more relaxed (n=3), less lonely (n=3), less depressed (n=3), more confident (n=3), more mentally stimulated (n=3) and energised (n=1)	7:<br>- 6 felt better<br>- 2 an opportunity to make a difference<br>- 2 feelings of freedom<br>- 1 feelings of independence and control	187	11 (control over daily life)	Joint second highest gain: Dignity/personal sense of significance<br><br>Joint third highest gain: Control over daily life
**Getting out of house / change of environment**	13<br>- getting out for a change of environment (n=10)<br>- somewhere to go (n=3)	6	46	*NOT APPLICABLE*	*NOT APPLICABLE*
**Practical support, information & access to other services**	18	-	47	*NOT APPLICABLE*	*NOT APPLICABLE*
**Physical wellbeing, health & safety**	9:<br>- informal health and wellbeing monitoring (n=4)<br>- increased exercise (n=4)<br>- feeling physically safe (n=1)	-	21	12 (personal safety)	Joint third highest gain: Personal safety
**Having a meal**	3	-	5	10 (food and drink)	Fourth highest gain: Food and drink
**Process outcomes**	3: sense of independence, freedom and control<br>Value for money (19)<br>15: good or very good value for money<br>1: reasonable value for money<br>1: not good value for money<br>2: unsure or value for money is irrelevant<br><br>23: plan to continue attending<br>23: would recommend DC to others (13 quite or very strongly)<br><br>16: Looks forward to or loves attending<br>3: Happy to or likes attending<br>2: Conflicting feelings<br>1: Makes self go – know is good for them<br>1: Used to love going	1: feelings of independence & control		11 (control over daily life)	Joint second highest gain: Dignity/personal sense of significance –
Accommodation cleanliness and comfort	*NOT APPLICABLE*	-	-	7	Lowest gain
Personal care	*NOT APPLICABLE*	-	-	7	No gain

Day centres for older people: attender characteristics, access routes and outcomes of regular attendance. Findings of exploratory mixed methods case study research.

Authors: Katharine Orellana, Jill Manthorpe, Anthea Tinker
